# Multiple snares-assisted endoscopic full-thickness resection of a giant submucosal tumor at the esophagogastric junction

**DOI:** 10.1055/a-2263-5991

**Published:** 2024-03-08

**Authors:** Jiyu Zhang, Miao Shi, Dan Liu, Lixia Zhao, Bingrong Liu

**Affiliations:** 1 191599 Department of Gastroenterology and Hepatology, The First Affiliated Hospital of Zhengzhou University, Zhengzhou, China

A 26-year-old woman presented to our hospital with intermittent dysphagia lasting 1 month. A contrast-enhanced computed tomography (CT) scan revealed a low-density accumulation at the esophagogastric junction, extending from the mediastinum to the abdominal cavity ( Fig. 1 **a** ). Endoscopic examination displayed a large submucosal bulge with a smooth mucosal surface, located 31 cm from the incisors ( Fig. 1 **b** ). Three-dimensional reconstruction techniques were utilized to assess the size and pinpoint the precise location of the lesion ( Fig. 1 **c** ). Endoscopic ultrasound confirmed its origin from the muscularis propria, showing hypoechoic changes ( Fig. 1 **d** ).

**Fig. 1:**
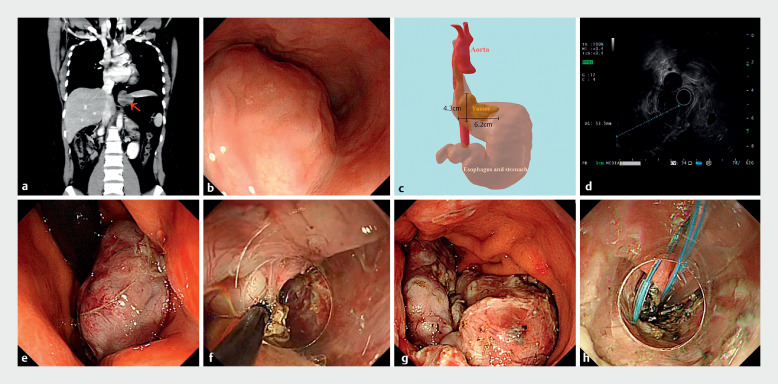
**a** The computed tomography (CT) scan revealed a low-density accumulation at the esophagogastric junction, extending from the mediastinum to the abdominal cavity. **b** An endoscopic examination displayed a large submucosal bulge with a smooth mucosal surface, located 31 cm from the incisors. **c** Three-dimensional reconstruction techniques were employed to assess the size and ascertain the precise location of the lesion. **d** Endoscopic ultrasound confirmed that the tumor originated from the muscularis propria, showing hypoechoic changes. **e** The esophageal segment of the lesion was detached from the muscularis propria, peeled off, and then pushed into the gastric cavity. **f** The remaining part of the lesion was completely excised with the help of snare assistance. **g** The excised lesion was segmented and extracted. **h** The surgical site was closed using the kissing suture technique.

Under general anesthesia, and following submucosal injection, an endoscopic incision was made in the lower esophagus using a HookKnife J (Olympus, Tokyo, Japan). The esophageal segment of the lesion was detached from the muscularis propria, peeled off, and pushed into the gastric cavity using snare assistance ( Fig. 1 **e** , Fig. 2 ). It was discovered that the gastric segment originated from the serosal layer of the gastric fundus. Another snare was then employed to lift the tumor, enhancing the visibility of the field and facilitating the complete resection of the remaining part ( Fig. 1 **f** , Video 1 ). Any bleeding encountered during the procedure was controlled. The excised lesion was segmented and removed ( Fig. 1 **g** ). Finally, the wound was sutured using the kissing suture method, and a gastrointestinal decompression tube was placed ( Fig. 1 **h** ). The pathological diagnosis was esophageal leiomyoma. No postoperative complications were observed, and the ulcer healed without perforation, as confirmed by endoscopy and CT examination 3 days post-procedure.

**Fig. 2:**
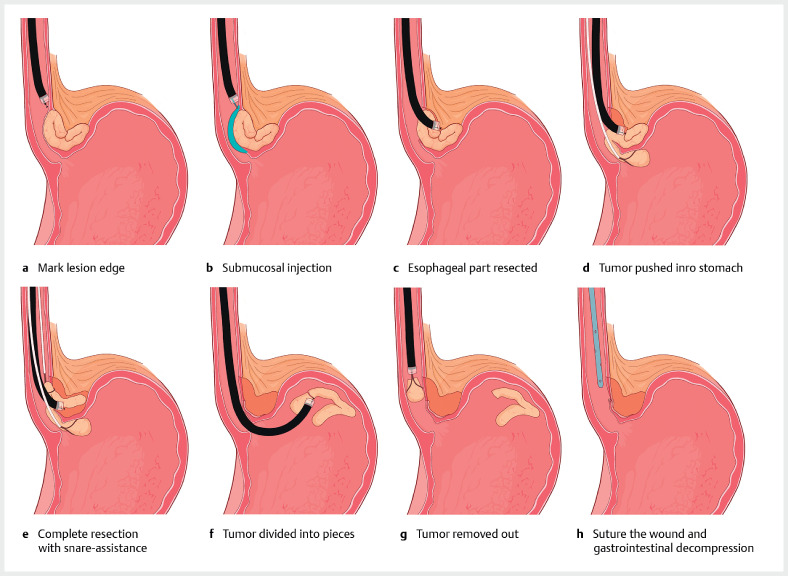
Schematic diagram of the procedure.

Multiple snare-assisted endoscopic full-thickness resection of a giant submucosal tumor at the esophagogastric junction.Video 1

The esophagogastric junction (EGJ) connects the esophagus to the stomach. Due to its unique anatomical structure, the EGJ has a narrow lumen with sharp angles, posing technical difficulties for endoscopic surgery and increasing the risk of complications like postoperative perforation. Endoscopic resection of submucosal tumors originating from the muscularis propria in this region is particularly challenging 1 . Additionally, preserving the functional integrity of the cardiac sphincter is crucial 2 3 . In this case, a multiple snare-assisted push-pull technique was employed, not only preserving the integrity of the cardia but also providing adequate space for complete tumor resection. We suggest that this innovative method offers a safe and effective approach for the resection of large submucosal tumors at the esophagogastric junction.

Endoscopy_UCTN_Code_TTT_1AO_2AG

## Notes

Endoscopy E-Videos https://eref.thieme.de/e-videos
*E-Videos* is an open access online section of the journal *Endoscopy* , reporting on interesting cases and new techniques in gastroenterological endoscopy. All papers include a high-quality video and are published with a Creative Commons CC-BY license. Endoscopy E-Videos qualify for HINARI discounts and waivers and eligibility is automatically checked during the submission process. We grant 100% waivers to articles whose corresponding authors are based in Group A countries and 50% waivers to those who are based in Group B countries as classified by Research4Life (see: https://www.research4life.org/access/eligibility/ ). This section has its own submission website at https://mc.manuscriptcentral.com/e-videos .
